# The outcome of acute kidney injury substages based on urinary cystatin C in critically ill children

**DOI:** 10.1186/s13613-023-01119-8

**Published:** 2023-03-28

**Authors:** Jiao Chen, Zhen Jiang, Hui Huang, Min Li, Zhenjiang Bai, Yuxian Kuai, Lin Wei, Ning Liu, Xiaozhong Li, Guoping Lu, Yanhong Li

**Affiliations:** 1 Department of Nephrology and Immunology, Children’s Hospital of Soochow University, Suzhou, Jiangsu Province China; 2 Pediatric Intensive Care Unit, Children’s Hospital of Soochow University, Suzhou, Jiangsu Province China; 3 Pediatric Intensive Care Unit, Xuzhou Children’s Hospital, Xuzhou, Jiangsu Province China; 4 Pediatric Intensive Care Unit, AnHui Provincial Children’s Hospital, Hefei, Anhui Province China; 5 Institute of Pediatric Research, Children’s Hospital of Soochow University, Suzhou, Jiangsu Province China; 6 Pediatric Intensive Care Unit, Children’s Hospital of Fudan University, Shanghai, China

**Keywords:** Acute kidney injury, Critically ill children, Mortality, Subclinical acute kidney injury, Urinary CysC

## Abstract

**Background:**

The concept of acute kidney injury (AKI) substages has been recommended to better phenotype AKI and identify high-risk patient groups and therefore improve the diagnostic accuracy of AKI. However, there remains a gap between the recommendation and the clinical application. The study aimed to explore the incidence of AKI substages based on a sensitive AKI biomarker of urinary cystatin C (uCysC), and to determine whether AKI substages were relevant with respect to outcome in critically ill children.

**Results:**

The multicenter cohort study enrolled 793 children in pediatric intensive care unit (PICU) of four tertiary hospitals in China. Children were classified as non-AKI, sub-AKI and AKI substages A and B according to uCysC level at PICU admission. Sub-AKI was defined by admission uCysC level ≥ 1.26 mg/g uCr in children not meeting the KDIGO criteria of AKI. In children who fulfilled KDIGO criteria, those with uCysC < 1.26 was defined as AKI substage A, and with ≥ 1.26 defined as AKI substage B. The associations of AKI substages with 30-day PICU mortality were assessed. 15.6% (124/793) of patients met the definition of sub-AKI. Of 180 (22.7%) patients with AKI, 90 (50%) had uCysC-positive AKI substage B and were more likely to have classical AKI stage 3, compared to substage A. Compared to non-AKI, sub-AKI and AKI substages A and B were risk factors significantly associated with mortality, and the association of sub-AKI (adjusted hazard ratio HR = 2.42) and AKI substage B (adjusted HR = 2.83) with mortality remained significant after adjustment for confounders. Moreover, AKI substage B had increased risks of death as compared with sub-AKI (HR = 3.10) and AKI substage A (HR = 3.19).

**Conclusions:**

Sub-AKI defined/based on uCysC occurred in 20.2% of patients without AKI and was associated with a risk of death close to patients with AKI substage A. Urinary CysC-positive AKI substage B occurred in 50% of AKI patients and was more likely to have classical AKI stage 3 and was associated with the highest risk of mortality.

**Supplementary Information:**

The online version contains supplementary material available at 10.1186/s13613-023-01119-8.

## Background

Acute kidney injury (AKI) remains associated with increased mortality in critically ill patients [1, 2]. Biomarker development has been recognized as a vital step toward initiating therapeutic intervention and potentially improving clinical outcomes [3–9]. The combination of damage and functional biomarkers, along with clinical information, has been recommended by the international consensus conference of Acute Dialysis Quality Initiative (ADQI) to be used to define AKI substages and identify high-risk patient groups and therefore to improve the diagnostic accuracy of AKI [8]. In AKI substages, the concept of subclinical AKI (sub-AKI) emerges to designate an episode of AKI unrecognized due to the absence of oliguria or rise in serum creatinine (SCr) level, and AKI substages A and B arise in attempting to subcategorize AKI stages by the absence or presence of biomarkers [3, 7, 8, 10–12]. However, there remains a gap between these recommendations and the clinical application, and whether elevation in biomarkers without any changes in SCr/oliguria or a change in SCr/oliguria without any change of damage biomarker is associated with worse kidney and patient outcomes remains elusive [7, 8, 13–15]. This is particularly true in children, because the results obtained in adults seem difficult to be transferred directly into the pediatric field, where the results and their interpretation are age-dependent [16, 17]. The incidence and outcome of AKI substages in critically ill children remain however largely unknown, and whether detecting AKI substages would be relevant with respect to outcome is needed to be investigated.

Cystatin C (CysC) is a low-molecular-weight protein and is freely filtered by the glomerulus. Increased level of urinary CysC (uCysC) may reflect renal tubular injury and impairment [18–20]. We have reported that uCysC, as a routine biochemical test, is independently predictive of AKI and mortality in critically ill neonates and children [21, 22], and derived the optimal cutoff value of 1.26 mg/g urine creatinine (uCr) of uCysC for the prediction [22]. Therefore, the aims of the prospective multicenter cohort study were to explore the incidence of AKI substages in critically ill children based on the cutoff value of uCysC, in conjunction with classical SCr/urine output, and to determine whether AKI substages were associated with adverse outcome in this population.

## Methods

### Study population

The study was conducted between September 2020 and February 2021 in the mixed PICUs of four tertiary teaching hospitals, including Children’s Hospital of Soochow University (Suzhou), Children’s Hospital of Fudan University (ShangHai), AnHui provincial Children’s Hospital (AnHui), and Xuzhou Children’s Hospital (Xuzhou). All children who met the criteria for PICU admission, adopted from guidelines for developing admission and discharge policies for the PICU [23], and had parental consent for participation were eligible for enrollment. The exclusion criteria were age of less than 1 month, known congenital abnormality of the kidney with abnormal kidney function, and failure to collect urine samples during the first day after PICU admission. The study was approved by the Institutional Review Board/Ethical Committee of the four hospitals and performed in accordance with the Declaration of Helsinki. Written informed parental consent was obtained at enrollment.

Upon admission, demographic and clinical data were recorded. Clinical status, comorbidities, and therapeutic intervention and medication including the length of mechanical ventilation (MV) and renal replacement therapy (RRT) were recorded until PICU discharge or death. The illness severity was assessed by the pediatric risk of mortality III (PRISM III) score, calculated based on the most abnormal values of physiological parameters collected in the first 24 h after PICU admission [24]. Sepsis was identified as children with confirmed or suspected infection with any pathogen who had acute organ dysfunction defined by a pediatric Sequential Organ Failure Assessment (pSOFA) score of 2 points or more by the treating physicians [25, 26]. Organ dysfunction, including respiratory dysfunction, circulatory dysfunction, coagulation dysfunction, hepatic failure, and multiple organ dysfunction syndrome (MODS) were diagnosed in accordance with the criteria by the international guidelines [25, 27].

### Diagnosis of AKI

The Kidney Disease: Improving Global Outcome (KDIGO) criteria for SCr and urine output were applied to define AKI and AKI stage developed during the PICU stay [28]. When the two criteria of SCr and urine output resulted in different AKI stages, the higher stage was chosen. The KDIGO stage 1 was defined as mild AKI, and KDIGO stage 2 or 3 was defined as severe AKI. The SCr level was routinely measured on PICU admission, followed by measurement every 48–72 h. The lowest SCr level within 3 months prior to admission was defined as baseline SCr. If baseline SCr was unavailable, the children were assumed to have an estimated glomerular filtration rate (eGFR) of 120 mL/min/1.73 m^2^ and the baseline SCr was back calculated according to the modified Schwartz formula [29]: eGFR = 0.413*height (cm)/SCr (mg/dL), in accordance with previous studies [1, 30, 31].

### AKI substages

Critically ill children were classified as non-AKI, sub-AKI, AKI substage A, and AKI substage B based on the combination of damage (uCysC) and functional (SCr or/and oliguria) biomarkers. The uCysC level ≥ 1.26 mg/g uCr indicates the presence of tubular injury [22]. Sub-AKI was defined by uCysC ≥ 1.26 mg/g uCr in critically ill children who did not meet the KDIGO criteria of AKI. In children who fulfilled the KDIGO criteria, AKI substage A was defined by uCysC < 1.26 mg/g uCr, and AKI substage B was defined by uCysC ≥ 1.26 mg/g uCr.

### Clinical outcome

The primary end point was 30-day PICU mortality defined as all-cause mortality occurring during the first 30 days after PICU admission, including death resulting from withdrawal of therapy.

### Urinary cystatin C measurements

The first available urine sample was collected on PICU admission using a plastic bag and immediately frozen and stored at − 80 °C. For the measurement, the samples with dry ice were delivered to the Children’s Hospital of Soochow University from other hospitals. In accordance with our previous study [22], samples were centrifuged and the supernatants were aliquoted and measured using an automatic biochemical analyzer (Hitachi 7600, Japan). The latex-enhanced immunoturbidimetry assay was used to measure uCysC, sarcosine oxidase method was used for uCr, and uCysC level was expressed in milligram per gram of uCr (mg/g uCr). The detection limit for uCysC was 10 ng/mL, and the intra-assay and inter-assay coefficients of variation were ≤ 10%, corresponding to that reported by the manufacturer. The samples with undetectable level of uCysC were assigned a uCysC value of 5 ng/mL, which is equivalent to half of the detection limit of the assay to facilitate the calculation of the ratio of uCysC to uCr.

### Statistical analysis

Continuous variables are expressed as median and interquartile range and were compared using Mann–Whitney *U* or Kruskal–Wallis tests after checking assumptions. Categorical variables are presented as number and percentage, and were compared using Chi-square analysis or Fisher exact tests. Univariable and multivariable logistic regression were performed to calculate the odds ratio (OR) and adjusted OR (AOR) with a 95% confidence interval (CI) to investigate the association between uCysC and AKI, and the uCsyC was included in the logistic regression as a dichotomous variable. To determine whether there exists multicollinearity among covariates, collinearity diagnostics was performed using variance inflation factor and tolerance values. Moreover, the non-AKI with the lowest risk of mortality was used as a reference, and Cox-regression analyses were performed to calculate the hazard ratio (HR) and adjusted HR to investigate whether sub-AKI and AKI substages A and B were independently associated with mortality after adjustment. All Cox-regression models included the covariate information in a time-dependent fashion. Kaplan–Meier survival analysis was used to describe cumulative 30-day PICU survival. The *P* value < 0.05 was considered statistically significant.

## Results

**Fig. 1:**
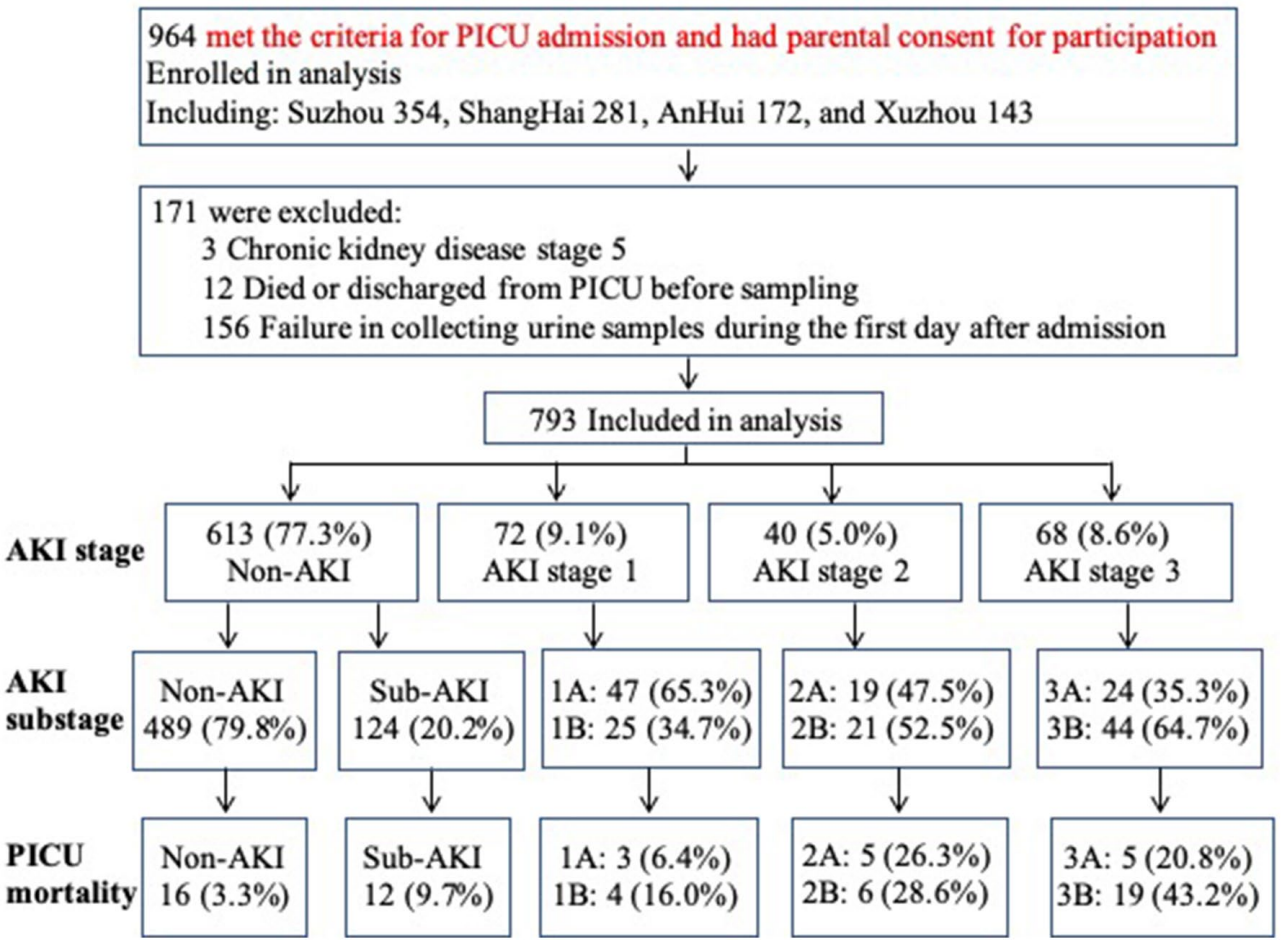
Flow diagram demonstrating study design. PICU, pediatric intensive care unit; sub-AKI, subclinical AKI

The prospective multicenter study involved 793 critically ill children between 1 month and 18 years of age from the PICUs of the four tertiary hospitals. Of the total of 964 children met the criteria for PICU admission and had parental consent for participation during the study period, 171 were excluded: 3 had chronic kidney disease stage 5, 12 died or were discharged from PICU before sampling, and 156 had a failure in collecting urine samples during the first day after admission (Fig. 1). Comparisons of the demographic and clinical characteristics between critically ill children included and excluded in the study are displayed in Additional file 1: Table S1.

The leading cause of PICU admission in the whole cohort was respiratory disease (23.8%), followed by neurologic disease (20.9%). Of the 793 children, 180 (22.7%) developed AKI, including 72 who fulfilled the criteria for KDIGO stage 1, 40 fulfilled stage 2, and 68 fulfilled stage 3, as shown in Fig. 1. All AKI children developed AKI during the first two weeks after admission, with 53.9% (*n* = 97) in the first day and 96.7% (*n* = 174) in the first week. The clinical characteristics among AKI status are displayed in Additional file 1: Table S2.

### Association between uCysC and AKI

**Fig. 2:**
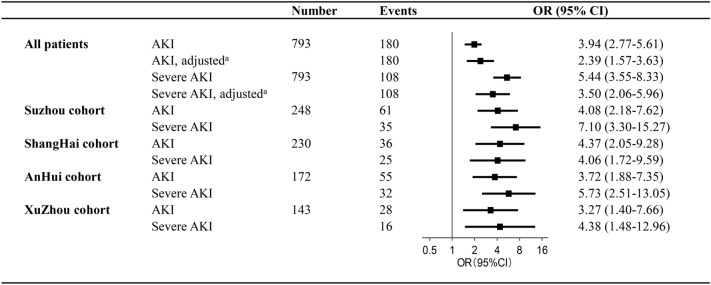
The risk of AKI and severe AKI on the basis of urinary cystatin C. AKI, acute kidney injury; CI, confidence interval; OR, odds ratio. Urinary cystatin C as a dichotomous variable based on the cutoff of 1.26 mg/g urinary creatinine. AKI developed during the pediatric intensive care unit stay was defined by KDIGO criteria. Severe AKI was defined as KDIGO stage 2 or 3. ^a^Adjusted for body weight, sex, illness severity assessed by the pediatric risk of mortality III score, sepsis, multi-organ dysfunction syndrome, shock/disseminated intravascular coagulation, and the use of mechanical ventilation, furosemide, and steroid

To determine whether uCysC, as a dichotomous variable based on the cutoff of 1.26 mg/g uCr, was independently associated with AKI, variables in Additional file 1: Table S2 were analyzed by the univariable logistic regression analysis, and those with a *P* < 0.05 were considered as confounding factors and entered into the multivariable regression after excluding multicollinear variables. As shown in Fig. 2, the uCysC was significantly associated with AKI (OR = 3.94, *P* < 0.001, *n* = 793), and the association between uCysC and AKI persisted in single center and remained significantly after adjustment for potential confounders including body weight, sex, illness severity assessed by PRISM III score, sepsis, MODS, shock/DIC, and the use of MV, furosemide, and steroid (AOR = 2.39, *P* < 0.001, *n* = 793). Similarly, the OR and AOR of uCysC at cutoff of 1.26 mg/g uCr for predicting severe AKI were 5.44 and 3.50, respectively (Fig. 2).

### AKI substages

**Fig. 3:**
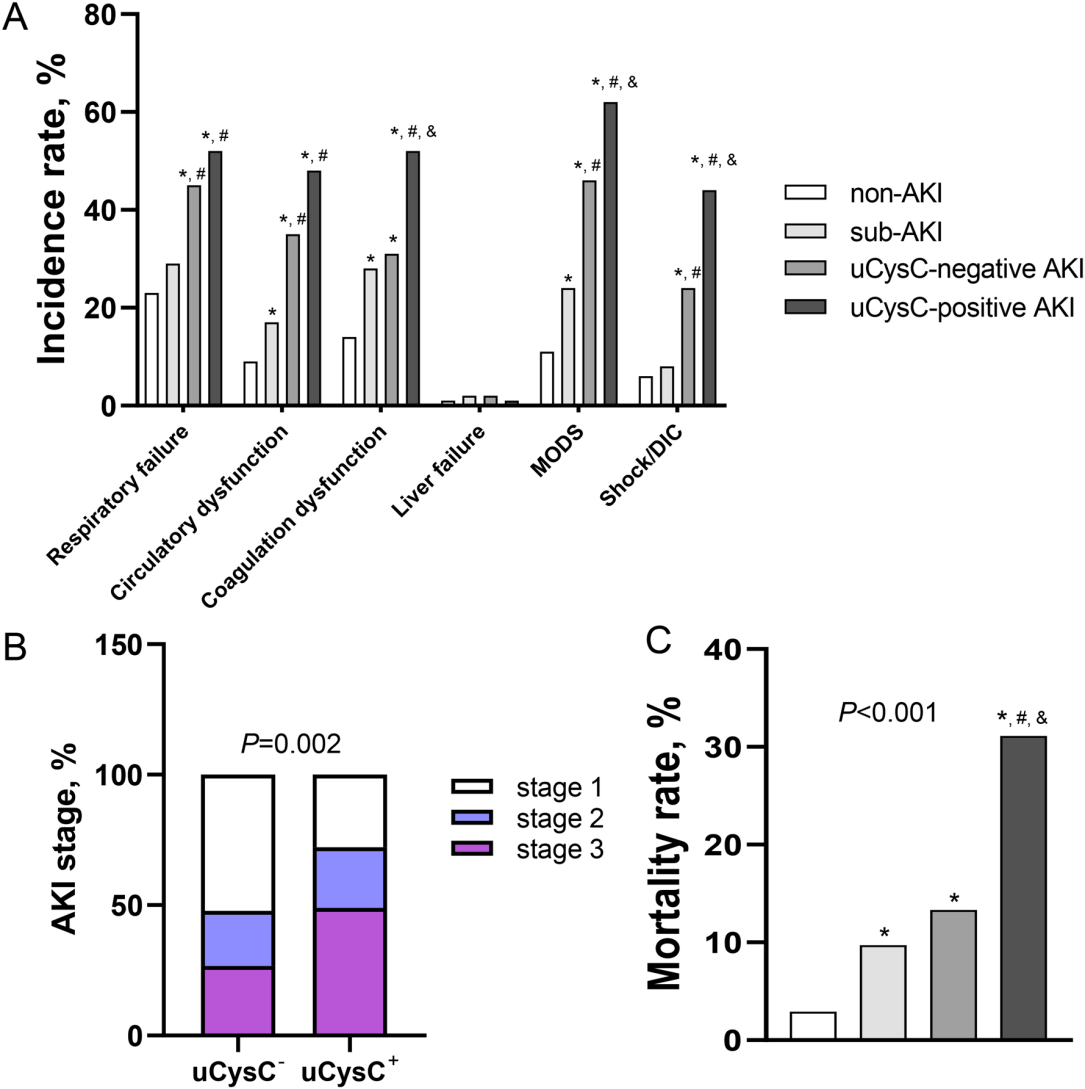
Comparison of the incidence rate. **A** Respiratory dysfunction (*P* < 0.001), circulatory dysfunction (*P* < 0.001), coagulation dysfunction (*P* < 0.001), hepatic failure (*P* = 0.889), multi-organ dysfunction syndrome (MODS) (*P* < 0.001), and shock/disseminated intravascular coagulation (DIC) (*P* < 0.001); **B** AKI stage between AKI substage A and B; **C** 30-day pediatric intensive care unit (PICU) mortality. AKI, acute kidney injury; sub-AKI, subclinical AKI; uCr, urinary creatinine; uCysC, urinary cystatin C. ^*^P < 0.05, vs. non-AKI; ^#^*P* < 0.05, vs. sub-AKI; ^&^*P* < 0.05, vs. AKI substage A

Critically ill children were classified as non-AKI, sub-AKI, AKI substage A, and AKI substage B. The incidence rates of non-AKI, sub-AKI, and AKI substages A and B in each stage of AKI defined by KDIGO criteria are displayed in Fig. 1. Of the 613 children without AKI, 124 had uCysC level ≥ 1.26 mg/g uCr and met the definition of sub-AKI. Of the 180 children with AKI, 90 had uCysC level < 1.26 mg/g uCr, which was defined as AKI substage A, and 90 had uCysC level ≥ 1.26 mg/g uCr, defined as AKI substage B. Demographic and clinical characteristics grouped according to these four groups are displayed in Table 1 and Fig. 3.

**Table 1:** Comparison of demographic and clinical characteristics among groups

	Non-AKI n = 613		AKI n = 180			*P* value
	Non-AKI	Subclinical AKI	AKI substage A		AKI substage B	
No. (%) with data	489 (61.7)	124 (15.6)	90 (11.3)		90 (11.3)	
Age, months	39.0 [12.0–88.0]	22.5 [5.0–52.8]^a^	23.5 [4.0–106.0]		14.0 [2.0–97.5]^a^	< 0.001
Body weight, kg	14.5 [9.5–25.3]	12.0 [6.8–16.9]^a^	13.0 [6.2–27.0]		10.0 [5.2–22.1]^a^	< 0.001
Male, n	298 (60.9)	75 (60.5)	59 (65.6)		52 (57.8)	0.841
PRISM III, score	3 [2–6]	7 [2–11]^a^	8 [2–12.3]^a^		12 [6.8–19.3]^a,b^^,c^	< 0.001
pSOFA, score	2 [1–4]	3 [1–5]^a^	5 [3–7]^a,b^		7 [4–12]^a,b^^,c^	< 0.001
Sepsis, n	49 (10.0)	20 (16.1)	22 (24.4)^a^		38 (42.2)^a,b^^,c^	< 0.001
MV, n	142 (29.0)	35 (28.2)	46 (51.1)^a,b^		52 (57.8)^a,b^	< 0.001
MV duration, hours	0 [0–5]	0 [0–31.5]	1 [0–158.8]^a^^,^^b^		23 [0–140.3]^a,b^	< 0.001
Inotrope, n	38 (7.8)	21 (16.9)^a^	30 (33.3)^a,b^		38 (42.2)^a,b^	< 0.001
Furosemide. n	130 (26.6)	37 (29.8)	54 (60.0)^a,b^		43 (47.8)^a^^,^^b^	< 0.001
Steroid, n	195 (39.9)	49 (39.5)	47 (52.2)^a^		35 (38.9)	0.158
PICU length of stay, day	5 (3–9)	6 (3–9)	7 (4–18.3)^a^^,^^b^		5 (2.8–10.3)^c^	0.001
PICU Mortality, n	16 (3.3)	12 (9.7)^a^	13 (14.4)^a^		29 (32.2)^a, b^^, c^	< 0.001
30-day PICU Mortality, n	14 (2.9)	12 (9.7)^a^	12 (13.3)^a^		28 (31.1)^a, b^^, c^	< 0.001

Values are median [interquartile range]. Numbers in parentheses denote percentages

AKI, acute kidney injury; MV, mechanical ventilation; PICU, pediatric intensive care unit; PRISM III, pediatric risk of mortality III; pSOFA, pediatric sequential organ failure assessment; uCr, urinary creatinine; uCysC, urinary cystatin C

AKI was defined by Kidney Disease: Improving Global Outcome (KDIGO) criteria. Subclinical AKI was defined by uCysC level ≥ 1.26 mg/g uCr in critically ill children who did not meet the KDIGO criteria of AKI. In critically ill children who fulfilled the KDIGO criteria of AKI, AKI substage A was defined by uCysC level < 1.26 mg/g uCr, and AKI substage B was defined by uCysC level ≥ 1.26 mg/g uCr

^a^*P* < 0.05, vs. non-AKI

^b^*P* < 0.05, vs. subclinical AKI

^c^*P* < 0.05, vs. AKI substage A

Sub-AKI was identified in 15.6% (124/793) of all critically ill children and in 20.2% (124/613) of those without AKI. In comparison to non-AKI children, those classified as sub-AKI were more likely to have circulatory dysfunction, coagulation dysfunction, and MODS (Fig. 3A). The AKI substage B was identified in 11.3% (90/793) of all children and in 50.0% (90/180) of those with AKI. In comparison to children with AKI substage A, children with AKI substage B were more likely to have coagulation dysfunction, MODS, and shock/DIC (Fig. 3A) and develop AKI stage 3 during the PICU stay (48.9% vs. 26.7%), as shown in Fig. 3B.

### AKI substages and 30-day PICU mortality

Comparisons of the clinical characteristics between survivors and non-survivors are displayed in Additional file 1: Table S3. The diagram in Fig. 1 displays the mortality rate of AKI substages. The 30-day PICU mortality rates in children with sub-AKI and AKI substages A and B were significantly higher than that in those with non-AKI, respectively (Fig. 3C). However, there was no difference in mortality between children with sub-AKI and those with AKI substage A (9.7% vs. 13.3%, *P* = 0.511). Mortality was significantly higher in children with AKI substage B than in those with sub-AKI (31.1% vs. 9.7%, *P* < 0.001) and in those with AKI substage A (31.1% vs. 13.3%, *P* = 0.007).

**Fig. 4:**
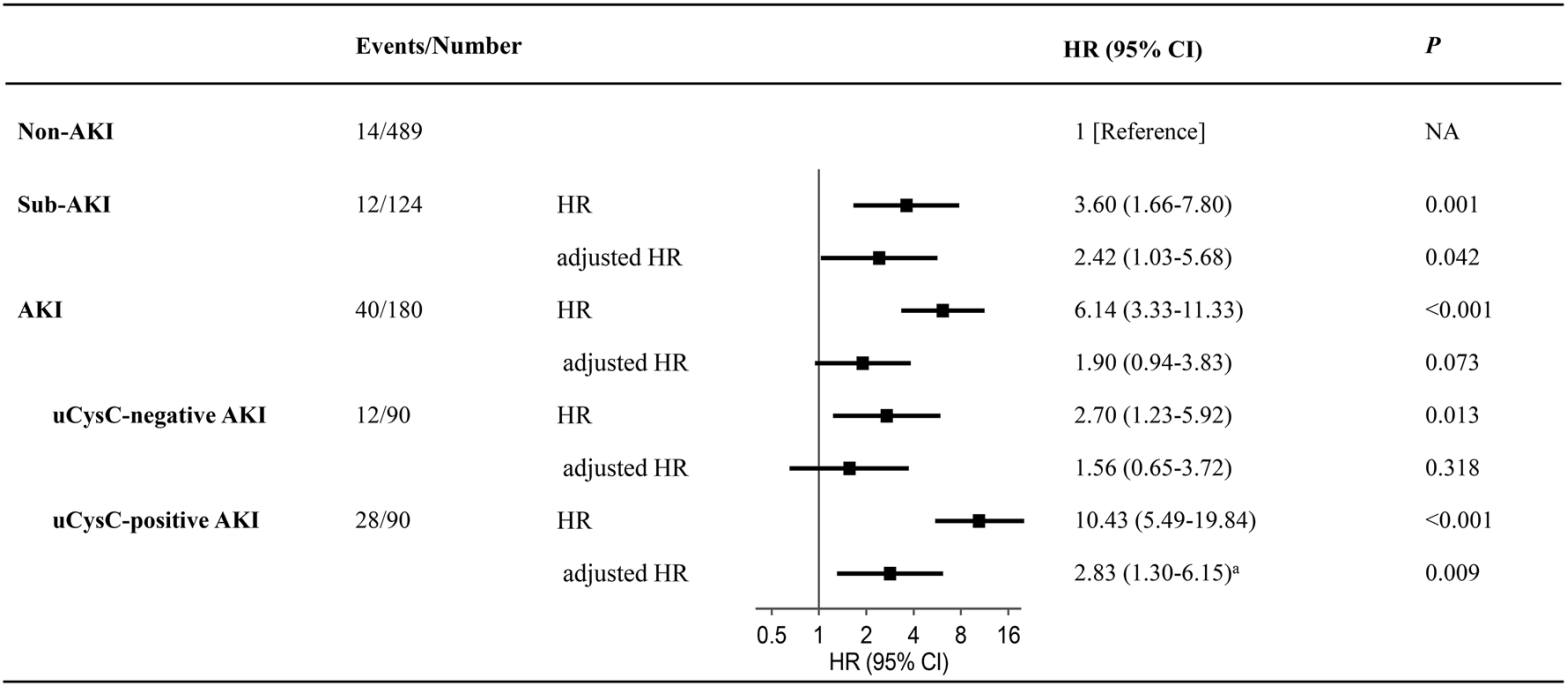
Forest plot depicting the risk of 30-day PICU mortality. The non-AKI with the lowest risk of mortality was used as a reference. Adjusted HR: Model was adjusted for body weight, sex, illness severity assessed by the pediatric risk of mortality III score, multi-organ dysfunction syndrome, and mechanical ventilation. KI, acute kidney injury; CI, confidence interval; HR, hazard ratio; NA, not applicable; PICU, pediatric intensive care unit; sub-AKI, subclinical AKI; uCr, urinary creatinine; uCysC, urinary cystatin C

**Fig. 5:**
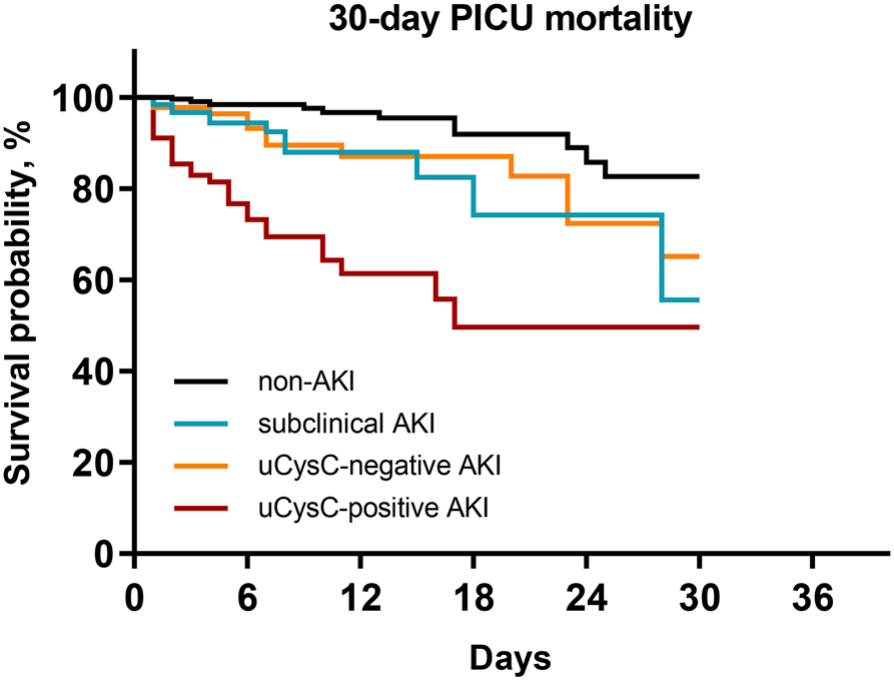
Kaplan–Meier survival curves of the four groups during the follow-up period of 30 days. Statistical significance was reached both when all four groups were considered (*P* < 0.001) and when only two groups (non-AKI vs. subclinical AKI, non-AKI vs. AKI substage A, non-AKI vs. AKI substage B, subclinical AKI vs. AKI substage B, AKI substage A vs. AKI substage B) were compared (*P* < 0.05)

Cox-regression analysis further revealed that as compared to non-AKI, sub-AKI and AKI substages A and B were risk factors significantly associated with 30-day PICU mortality (Fig. 4). The association of sub-AKI (adjusted HR = 2.42, *P* = 0.042, *n* = 613) and AKI substage B (adjusted HR = 2.83, *P* = 0.009, *n* = 579), but not AKI substage A (adjusted HR = 1.56, *P* = 0.318, *n* = 579), with mortality remained significant after adjustment for body weight, sex, PRISM III score, MODS, and MV in Cox-regression models. Moreover, AKI substage B was significantly associated with a higher risk of mortality, when compared to sub-AKI (HR = 3.10, 95% CI 1.58–6.11, *P* = 0.001, *n* = 214) and AKI substage A (HR = 3.19, 95% CI 1.60–6.33, *P* = 0.001, *n* = 180), respectively. Findings were consistent in a time-to-event analysis in Fig. 5.

## Discussion

The study is the first known attempt to explore the outcome of AKI substages based on urinary CysC in critically ill children. Among PICU patients not meeting the KDIGO criteria of AKI, sub-AKI determined by uCysC concentration at admission was associated with increased risk for mortality. Urinary CysC-positive AKI substage B demonstrated the greatest discriminative power for subsequent development of mortality. Our data imply that the combined use of damage and functional biomarkers can be used to defined AKI subtypes and identify high-risk patient groups in critically ill children.

This study assessed the predictive accuracy of uCysC at the cutoff of 1.26 mg/g uCr, and clearly showed that the uCysC at the threshold can be used to identify greater risk of AKI in critically ill children at the age of more than 1 month. As we know, defining an efficient and reliable tool for AKI assessment in children is still a challenge, because the pathophysiology of AKI in children can be affected by ongoing tubular development, greater renal reserve, and superior renal regenerative potential compared with adults [16, 17, 32]. Although as a sensitive biomarker for AKI and mortality [20–22, 33, 34], the levels of uCysC are inversely correlated with age and body weight in neonates and children [21, 22, 35, 36]. This multicenter cohort study with a large general and heterogeneous pediatric population provides adequate power for validating threshold and increases the generalizability of our findings. Our data provide a valuable measure to refine the current definition of AKI and supply evidence that uCysC at the cutoff of 1.26 is not only helpful in the discrimination of AKI, but also indicative of AKI subtypes. Potential utility of uCysC is underlined by the proof of a prognostic impact associated with AKI subtypes.

The combination of damage and functional biomarkers can be used to identify an early stage when there is evidence of kidney injury that is not detected by SCr and urine output criteria. The uCysC threshold of 1.26 mg/g uCr identified 15.6% of critically ill children with sub-AKI, allowing better phenotyping of critically ill children previously classified as non-AKI. The mortality in children with sub-AKI was higher than in children with non-AKI and close to children with uCysC-negative AKI substage A. In addition, the association of sub-AKI with increased mortality persisted when accounting for potential confounders, including illness severity. These results are consistent with recent reports conducted in adult patients, showing that sub-AKI defined using plasma or urinary biomarkers was associated with a risk of death close to patients with AKI [11, 37, 38]. Our findings endorse the notion that sub-AKI is an early subtype of AKI, and regardless of the exact pathophysiology of these patients, given the trend towards poor outcomes, detecting this subtype is clinically significant and may guide pediatricians in their clinical decision-making and their stepwise approach to the complex management of critically ill children requiring timing interventions in the course of critical illness.

As a precise and stable routine test [39] to diagnose AKI, the impact of the measurement of uCysC was also pronounced within the cohort of critically ill children with classically defined AKI. The other major finding in the study was to integrate uCysC with SCr/oliguria to delineate AKI phenotypes in critically ill children. Half of the children with traditionally defined KDIGO AKI were found to have no biomarker evidence of tubular injury but have loss of kidney function and were subcategorized to substage A. Decreased glomerular filtration without renal tubule injury may represent a state of pre-renal azotemia, which is considered a volume-responsive, reversible alteration in kidney function. Indeed, when compared with those with AKI substage A, children with AKI substage B had biomarker evidence of tubular injury and were more likely to have classical AKI stage 3 during the PICU stay and have increased mortality rate regardless of the stage of AKI. Most powerfully, when examining mortality, relative to children with AKI substage A, AKI children with substage B were associated with increased mortality in multivariable analysis, suggesting that the impact of the AKI phenotype was most pronounced within the cohort of children with AKI substage B. Our results are consistent with studies conducted in adult patients and in critically ill children [40, 41]. In adult patients with cardiorenal syndrome, urine neutrophil gelatinase-associated lipocalin (NGAL) positive AKI had the greatest risk of mortality [40]. Similarly, compared with critically ill children with elevated urinary NGAL concentrations without increased SCr levels, those with urinary NGAL and SCr increases had a 12-fold increased risk of AKI stage 2 or 3 [42]. The findings demonstrate that not all standardly staged AKI is equivalent and associated outcome varies within patients with different AKI subtypes. The combination of damage (uCysC at the cutoff point of 1.26) and functional (SCr/urine output) biomarkers was superior to that used alone in predicting AKI severity and worse outcomes and has the potential to provide important information to determine dosing and duration of personalized treatment and to prevent adverse outcomes.

The pathophysiologic mechanisms underlying the independent association between AKI substages and mortality in critically ill children remain to be elucidated. Although the differences in outcomes between the AKI substages may be related to the severity of illness, all analyses in the study adjusted for illness severity, and the substages are consistently associated with different mortality risks. We speculate that uncharacterized clinically and physiologically distinct substages exist within critically ill children with AKI and that they are associated with differences in the risk of adverse outcomes. Moreover, because of the inherent heterogeneity of AKI in its etiology and pathophysiology, human studies investigating the prevention and treatment of AKI remain largely inconclusive [43, 44]. The identification of AKI subphenotypes linked to underlying pathophysiologic processes may parse the heterogeneity in the AKI phenotype and identify treatment-responsive AKI subtypes [45, 46]. Further studies in large cohorts are necessary to determine whether the AKI substages identified by the biomarker are associated with distinct clinical characteristics and have therapeutic implications to target more precisely patients who are responsive to specific therapies.

There are limitations to our study. First, SCr was not measured daily as part of the study, and AKI patients might be under-detected. Next, the majority of children did not have baseline SCr prior to admission, and we calculated baseline SCr using the modified Schwartz formula in accordance to previous studies [1, 30, 31]. However, it remains uncertain how to best determine baseline kidney function [47] and the accuracy of these equations, including the one used in the study, has not been validated in heterogeneous PICU population. Third, our results might be biased by the fact that 156 patients (16.2%) were not included due to the absence of collection of urine samples in the first 24 h. Although there was no significant difference in the incidence and severity distribution of AKI or mortality rate between included and excluded patients, the excluded patients tended to be a female and less severe with a lower PRISM III score, and only 10.2% of the excluded patients developed MODS and 13.9% had been mechanically ventilated during the PICU stay, which might bias the study towards positive results. Nevertheless, our results are strengthened by the fact that the association of urinary CysC-positive AKI substages with mortality was independent of sex, illness severity, the presence of MODS, and the use of MV. Fourth, we did not compare uCysC to other biomarkers of renal damage (e.g., NGAL, kidney injury molecule-1, or liver-type fatty acid-binding protein). Whether or not the combination of uCysC and other biomarkers further improve the ability in defining AKI phenotypes needs to be explored.

## Conclusion

The uCysC cutoff of 1.26 mg/g uCr determined at PICU admission is a valuable threshold for AKI risk assessment to define AKI subtypes. Sub-AKI defined/based on uCysC occurred in 20.2% of critically ill children without AKI and was associated with a risk of death close to patients with AKI substage A. Urinary CysC-positive AKI substage B occurred in 50% of AKI children and was more likely to have classical AKI stage 3 and was associated with the highest risk of mortality. Further randomized clinical trials are needed to explore whether timely and personalized intervention based on AKI subtypes would improve the clinical outcomes in critically ill children.

## Supplementary Information

**Additional file 1: Table S1.** Comparison of demographic and clinical characteristics between critically ill children included and excluded in the study **Table S2.** Comparison of demographic and clinical characteristics among AKI status **Table S3.**Comparison of demographic and clinical characteristics between 30-day PICU survivors and non-survivors

## Data Availability

The datasets used and/or analyzed during the current study are available from the corresponding author on reasonable request.

## Abbreviations

AKI: Acute kidney injury
ADQI: Acute Dialysis Quality Initiative
AOR: Adjusted odds ratio
AUC: Area under the receiver operating characteristic curve
CI: Confidence interval
Cr: Creatinine
CysC: Cystatin C
DIC: Disseminated intravascular coagulation
eGFR: Estimated glomerular filtration rate
HR: Hazard ratio
IQR: Interquartile range
KDIGO: Kidney Disease: Improving Global Outcomes
LOS: Length of stay
MODS: Multiple organ dysfunction syndrome
MV: Mechanical ventilation
NGAL: Neutrophil gelatinase-associated lipocalin
OR: Odds ratio
PICU: Pediatric intensive care unit
PRISM III: Pediatric risk of mortality III
pSOFA: Pediatric sequential organ failure assessment
ROC: Receiver operating characteristic curve
RRT: Renal replacement therapy
SCr: Serum creatinine
sub-AKI: Subclinical AKI
uCysC: Urinary cystatin C
uCr: Urine creatinine
