# Supplementary material for: The outcome of acute kidney injury substages based on urinary cystatin C in critically ill children
Source: Ann Intensive Care. 2023 Mar 28;13:23. doi: 10.1186/s13613-023-01119-8 (PMC10050666; doi:10.1186/s13613-023-01119-8)
Supplement: Supplementary file 1 — Additional file 1: Table S1. Comparison of demographic and clinical characteristics between critically ill children included and excluded in the study Table S2. Comparison of demographic and clinical characteristics among AKI status Table S3.Comparison of demographic and clinical characteristics between 30-day PICU survivors and non-survivors [file 13613_2023_1119_MOESM1_ESM.docx]

Table S1. Comparison of demographic and clinical characteristics between critically ill children included and excluded in the study

|  | **Included, n=793** | **Excluded, n=171** | ***P* value** |
| --- | --- | --- | --- |
| Age, months | 32.0 [9.0-84.0] | 34.0 [10.0-98.8] | 0.309 |
| Body weight, kg | 13.0 [8.3-24.0] | 14.1 [9.0-26.0] | 0.239 |
| Male, n | 483 (60.9) | 89 (52.0) | 0.039 |
| PRISM III, score | 5 [2-10] | 4 [2-7] | 0.039 |
| pSOFA, score | 3 [1-5] | 2 [1-4] | 0.002 |
| Sepsis, n | 129 (16.3) | 28 (16.4) | 0.973 |
| AKI, n | 180 (22.7) | 36 (21.1) | 0.687 |
| AKI stage 1, n | 72 (9.1) | 20 (11.7) | 0.361 |
| AKI stage 2, n | 40 (5.0) | 5 (2.9) |  |
| AKI stage 3, n | 68 (8.6) | 11 (6.4) |  |
| MODS | 184 (23.2) | 19 (11.1) | <0.001 |
| Shock/DIC | 106 (13.4) | 22 (12.9) | 0.902 |
| MV, n | 275 (34.7) | 23 (13.5) | <0.001 |
| MV duration^a^, hours | 115 [39-242] | 117 [24-229] | 0.865 |
| PICU length of stay, day | 5 [3-10] | 3 [1-6] | <0.001 |
| PICU Mortality, n | 70 (8.8) | 9 (5.3) | 0.128 |

Values are median [interquartile range]. Numbers in parentheses denote percentages.

AKI, acute kidney injury; DIC, disseminated intravascular coagulation; MODS, multi-organ dysfunction syndrome; MV, mechanical ventilation; PICU, pediatric intensive care unit; PRISM III, pediatric risk of mortality III; pSOFA, pediatric sequential organ failure assessment

^a^In patients with mechanical ventilation.

Table S2. Comparison of demographic and clinical characteristics among AKI status

|  | **Non-AKI** | **AKI stage 1** | **AKI stage 2** | **AKI stage 3** | ***P* value** |
| --- | --- | --- | --- | --- | --- |
| No. (%) | 613 (77.3) | 72 (9.1) | 40 (5.0) | 68 (8.6) |  |
| Age, months | 34 (11-84) | 11.5 (2-54.3)^a^ | 15.5 (3.3-94.5) | 50.5 (8.3-120)^b^ | <0.001 |
| Body weight, kg | 13.5 (9.0-24.0) | 9.4 (5.1-17.1)^a^ | 9.0 (5.4-25.0)^a^ | 15.3(8.5-36.4)^b, c^ | <0.001 |
| Male, n | 373 (60.8) | 43 (59.7) | 28 (70.0) | 39 (57.4) | 0.616 |
| PRISM III, score | 3 (2-7.5) | 8 (2-11)^a^ | 8 (2-18)^a^ | 12.5 (9-21)^a, b, c^ | <0.001 |
| pSOFA, score | 2 (1-4) | 4 (2-6)^a^ | 6 (4-11)^a, b^ | 8 (5-12)^a, b, c^ | <0.001 |
| Sepsis, n | 69 (11.3) | 14 (19.4) | 13 (32.5)^a^ | 33 (48.5)^a, b^ | <0.001 |
| Respiratory failure, n | 154 (25.1) | 27 (37.5)^a^ | 26 (65.0)^a, b^ | 35 (51.5)^a^ | <0.001 |
| Circulatory dysfunction, n | 67 (10.9) | 19 (26.4)^a^ | 15 (37.5)^a^ | 42 (61.8)^a, b, c^ | <0.001 |
| Coagulation dysfunction, n | 107 (17.5) | 18 (25.0) | 16 (40.0)^a^ | 40 (58.8)^a, b^ | <0.001 |
| Hepatic failure, n | 11 (1.8) | 0 (0.0) | 1 (2.5) | 2 (2.9) | 0.398 |
| MODS, n | 86 (14.0) | 21 (29.2)^a^ | 25 (62.5)^a, b^ | 52 (76.5)^a, b^ | <0.001 |
| Shock/DIC, n | 44 (7.2) | 13 (18.1)^a^ | 13 (32.5)^a^ | 36 (52.9)^a, b, c^ | <0.001 |
| MV, n | 177 (28.9) | 29 (40.3) | 27 (67.5)^a, b^ | 42 (61.8)^a, b^ | <0.001 |
| MV Duration, hour | 0 (0-7) | 0 (0-133.5)^a^ | 30.5 (0-126.3)^a^ | 31.5 (0-204)^a^ | <0.001 |
| Inotrope, n | 59 (9.6) | 12 (16.7) | 18 (45.0)^a, b^ | 38 (55.9)^a, b^ | <.001 |
| Furosemide. n | 167 (27.2) | 31 (43.1)^a^ | 24 (60.0)^a^ | 41 (60.3)^a, b^ | <0.001 |
| Steroid, n | 244 (39.8) | 24 (33.3) | 19 (47.5) | 39 (57.4)^b^ | 0.015 |
| PICU length of stay, day | 5 (3-9) | 5 (3-14) | 6 (3-9.8) | 7 (5-15.8)^a, c^ | 0.002 |
| PICU Morality, n | 28 (4.6) | 7 (9.7) | 11 (27.5)^a, b^ | 24 (35.3)^a, b^ | <0.001 |
| 30-day PICU Morality, n | 26 (4.2) | 6 (8.3) | 11 (27.5)^a, b^ | 23 (33.8)^a, b^ | <0.001 |
| uCysC, mg/g uCr | 0.50  [0.23-1.11] | 0.81  [0.34-2.26]^a^ | 1.67  [0.57-8.79]^a, b^ | 3.15  [0.63-16.64]^a, b^ | <0.001 |
| Suzhou cohort, n=248 | 0.37  [0.19-0.98] | 0.70  [0.38-1.50]^a^ | 1.46  [0.59-14.68]^a, b^ | 2.69  [0.55-15.01]^a, b^ | <0.001 |
| ShangHai cohort, n=230 | 0.50  [0.23-1.02] | 0.79  [0.27-44.73] | 0.65  [0.43-2.12] | 2.81  [0.67-13.64]^a^ | <0.001 |
| AnHui cohort, n=172 | 0.50  [0.21-1.25] | 0.94 [0.23-2.99] | 2.49 [0.63-13.12]^a, b^ | 3.44  [0.95-30.03]^a, b^ | <0.001 |
| XuZhou cohort, n=143 | 0.63  [0.33-1.39] | 0.81  [0.43-4.18] | 1.27  [0.29-8.42] | 6.68  [0.87-26.93]^a^ | 0.029 |

Values are median [interquartile range]. Numbers in parentheses denote percentages.

AKI, acute kidney injury; DIC, disseminated intravascular coagulation; MODS, multi-organ dysfunction syndrome; MV, mechanical ventilation; PICU, pediatric intensive care unit; PRISM III, pediatric risk of mortality III; pSOFA, pediatric sequential organ failure assessment; uCr, urinary creatinine; uCysC, urinary cystatin-C.

AKI developed during the PICU stay was defined by Kidney Disease: Improving Global Outcome (KDIGO) criteria.

^a^*P*<0.05, vs. non-AKI; ^b^*P*<0.05, vs. AKI stage 1; ^c^*P*<0.05, vs. AKI stage 2.

Table S3. Comparison of demographic and clinical characteristics between 30-day PICU survivors and non-survivors

|  | **Survivors** | **Non-survivors** | ***P* value** |
| --- | --- | --- | --- |
| No. (%) | 727 (91.7) | 66 (8.3) |  |
| Age, months | 32.0 (9.0-84.0) | 40.0 (10.8-106.8) | 0.183 |
| Body weight, kg | 13.0 (8.1-24.0) | 14.0 (8.9-25.5) | 0.442 |
| Male, n | 452 (62.2) | 31 (47.0) | 0.018 |
| PRISM III, score | 4 (2-8) | 14.5 (8-24) | <0.001 |
| pSOFA, score | 3 (1-4) | 9 (6-12) | <0.001 |
| Sepsis, n | 103 (14.2) | 26 (39.4) | <0.001 |
| AKI, n | 140 (19.3) | 40 (60.6) | <0.001 |
| AKI stage 1, n | 66 (9.1) | 6 (9.1) | <0.001 |
| AKI stage 2, n | 29 (4.0) | 11 (16.7) |  |
| AKI stage 3, n | 45 (6.2) | 23 (34.8) |  |
| Respiratory failure, n | 196 (27.0) | 46 (69.7) | <0.001 |
| Circulatory dysfunction, n | 99 (13.6) | 44 (66.7) | <0.001 |
| Coagulation dysfunction, n | 136 (18.7) | 45 (68.2) | <0.001 |
| Hepatic failure, n | 11 (1.5) | 3 (4.5) | 0.396 |
| MODS, n | 130 (17.9) | 54 (81.8) | <0.001 |
| Shock/DIC, n | 68 (9.4) | 38 (57.6) | <0.001 |
| MV, n | 218 (30.0) | 57 (86.4) | <0.001 |
| MV Duration, hour | 0 (0-22) | 56.5 (19-194) | <0.001 |
| Inotrope, n | 81 (11.1) | 46 (69.7) | <0.001 |
| Furosemide. n | 223 (30.7) | 40 (60.6) | <0.001 |
| Steroid, n | 297 (40.9) | 29 (43.9) | 0.695 |
| PICU length of stay, day | 5 (3-9) | 5 (2-11) | 0.568 |
| uCysC, mg/g uCr | 0.55 (0.25-1.26) | 2.07 (0.55-14.81) | <0.001 |
| Suzhou cohort, n=248 | 0.47 (0.23-1.14) | 2.03 (0.57-19.53) | <0.001 |
| ShangHai cohort, n=230 | 0.54 (0.24-1.09) | 1.28 (0.34-3.02) | <0.001 |
| AnHui cohort, n=172 | 0.57 (0.23-1.73) | 4.49 (0.39-32.90) | <0.001 |
| XuZhou cohort, n=143 | 0.69 (0.34-1.66) | 1.98 (0.53-9.52) | <0.001 |

Values are median [interquartile range]. Numbers in parentheses denote percentages.

AKI, acute kidney injury; DIC, disseminated intravascular coagulation; MODS, multi-organ dysfunction syndrome; MV, mechanical ventilation; PICU, pediatric intensive care unit; PRISM III, pediatric risk of mortality III; pSOFA, pediatric sequential organ failure assessment; uCr, urinary creatinine; uCysC, urinary cystatin-C.
